# Multiple sequence alignment: a major challenge to large-scale phylogenetics

**DOI:** 10.1371/currents.RRN1198

**Published:** 2011-04-07

**Authors:** Kevin Liu, C. Randal Linder, Tandy Warnow

**Affiliations:** ^*^The University of Texas; ^†^Integrative Biology, University of Texas and ^‡^Microsoft Research New England, and The Department of Computer Science, University of Texas at Austin

## Abstract

Over the last decade, dramatic advances have been made in developing methods for large-scale phylogeny estimation, so that it is now feasible for investigators with moderate computational resources to obtain reasonable solutions to maximum likelihood and maximum parsimony, even for datasets with a few thousand sequences. There has also been progress on developing methods for multiple sequence alignment, so that greater alignment accuracy (and subsequent improvement in phylogenetic accuracy) is now possible through automated methods. However, these methods have not been tested under conditions that reflect properties of datasets confronted by large-scale phylogenetic estimation projects. In this paper we report on a study that compares several alignment methods on a benchmark collection of nucleotide sequence datasets of up to 78,132 sequences. We show that as the number of sequences increases, the number of alignment methods that can analyze the datasets decreases. Furthermore, the most accurate alignment methods are unable to analyze the very largest datasets we studied, so that only moderately accurate alignment methods can be used on the largest datasets. As a result, alignments computed for large datasets have relatively large error rates, and maximum likelihood phylogenies computed on these alignments also have high error rates. Therefore, the estimation of highly accurate multiple sequence alignments is a major challenge for Tree of Life projects, and more generally for large-scale systematics studies.

## **1  Introduction**

    Species trees are typically estimated from a collection of genes (or other genomic regions), in one of two ways.  The first way produces alignments on each gene, and then runs a phylogeny estimation method on the concatenation of these alignments; the second way estimates trees for each gene based upon an alignment of the sequences, and then combines these estimated gene trees into a species tree. In both cases, therefore, the accuracy of the resultant species tree depends directly or indirectly upon the alignments that are produced for each gene [1] [2] [3] [4] [5] [6] [7].  Because of the centrality of sequence alignment to phylogenetics (and other problems in biology), many alignment methods have been developed.  ClustalW [8] is perhaps the most well known, and probably the most frequently used alignment method in systematics, but there are many others, including MAFFT [9], T-Coffee [10], Probcons [11], POY [12], and Muscle [13], that are used in the systematics community. There are also newer methods, i.e., Opal [14], Prank [15], Prank+GT [16], FSA [17], POY* [18], SATCHMO [19], ProbAlign [20], ProbTree [21], BALi-Phy [22], and SATé [16], which have also been developed but are less frequently used.

    Evaluations of these methods on both simulated and biological datasets show that alignment accuracy impacts tree accuracy, and that some methods (notably MAFFT and SATé) can produce highly accurate alignments on large datasets, and hence make it possible to construct highly accurate trees when trees are computed using maximum likelihood (ML) [7] [16]. However, these evaluations have been largely limited to datasets containing at most 1000 sequences, and so are not necessarily relevant to large-scale systematics studies.

    In this paper, we explore the performance of alignment methods on a collection of nucleotide datasets containing large numbers of sequences. We report computational requirements, including running time and memory usage, and also the accuracy of the alignments and of maximum likelihood (ML) trees estimated on these alignments.

   These comparisons show striking differences between methods. Alignment methods differ in their computational requirements, with some methods incapable of analyzing datasets beyond a few hundred sequences, and others able to analyze datasets with tens of thousands of sequences. Running time and memory usage for some alignment methods can be enormous, sometimes exceeding the computational requirements of RAxML [23] [24], currently one of the most frequently used methods for large-scale phylogenetic ML estimation. Methods also differ substantially with respect to alignment and subsequent tree accuracy, so that the selection of alignment method is very important to the systematics study.

    The main observation in this study is that only a small number of multiple sequence alignment methods can be run on datasets with several thousand sequences. Furthermore, of those methods that can be run on large datasets, none produces alignments of sufficient accuracy to be useful in estimating highly accurate phylogenies.  Therefore, when datasets are large and have evolved with many indels, the input to species tree estimations (i.e. either super-alignments or  gene trees) are likely to be of poor accuracy; consequently, this suggests that species tree estimates will also have reduced accuracy for large datasets, directly because of alignment difficulties. 

## **2  Methodology**

### 2.1  Overview

We used several rRNA biological datasets (of up to 27,643 sequences) and one simulated dataset (of 78,132 sequences) to evaluate the alignment methods. The biological datasets have curated alignments based upon secondary structure and reference trees produced by computing maximum likelihood trees with bootstrapping on each curated alignment, retaining only the high support edges. For the simulated dataset, we have the true alignment and true tree. See Table 1 and the supplementary online materials [25] for details about these data.

    For each dataset, we attempted to compute alignments using a collection of alignment methods, and for each alignment that we generated, we computed a maximum likelihood tree using either RAxML or FastTree [26] [27]. We compared the estimated alignments to the true or curated alignment to determine the alignment error rate, and we compared the estimated ML tree to the true or reference tree to determine the tree error rate. We also recorded running time for each analysis.

## 2.2  Datasets

The biological datasets were drawn from Robin Gutell’s Comparative RNA Website (CRW) [28] and have curated alignments based upon secondary structure. While secondary structure alignments are highly reliable, there is no guarantee that these are perfectly correct.  However, these are the most reliable benchmarks available to date for testing nucleotide alignment methods, other than using simulated data. These biological datasets contain 16S and 23S sequences, markers that are frequently used for estimating phylogenies. They range in size from about 100 sequences to almost 28,000 sequences. We used the preprocessing steps in [16] on these datasets to remove taxa that were more than 50% unsequenced, and to remove illegal characters. The empirical statistics for the resulting curated alignments after preprocessing are listed in Table 1. Note that these biological datasets are relatively “gappy", with indels occupying 60-90% of the curated alignment matrix. They also have different gap length distributions, and different average and maximum p-distances. Thus, these datasets have properties that are challenging for alignment and phylogeny estimation, and are realistic examples of datasets used in large-scale phylogenetic studies. See the Supplementary Materials online webpage [25] for these data.

    The simulated dataset was obtained from [27], with 78,132 DNA sequences and 1,287 sites in the true alignment. This dataset is the last replicate from the simulation by [27], obtained at http://www.microbesonline.org/fasttree/; we call this the “Price 78K" dataset. The empirical statistics for the true alignment on this dataset are in Table 1. Many of the empirical statistics for the Price 78K dataset are similar to those of the empirical datasets; however, the Price 78K dataset has hardly any indels (in fact the gappiness is two orders of magnitude less than those exhibited in the biological datasets) and the average gap length is extremely short.  As a result, the Price 78K dataset is potentially easier to align than the biological datasets.  Because of its size, however, the Price 78K dataset provides a test of scalability.

    Reference alignments and trees were computed as follows. For the Price 78K dataset, we used the true alignment as the reference alignment, and the true tree as the reference tree; these are known because the Price 78K dataset is simulated. For the biological datasets, the curated alignment, modified by our minor cleaning operations, is the reference alignment. We computed unaligned sequences from the reference alignments by simply deleting indels (“-”). To obtain reference trees for the biological datasets, we performed rapid bootstrapping analyses with RAxML [29] on the curated alignment to produce a tree with support values, and then contracted all edges with less than 75% support. We generated 500 bootstrap replicates on all datasets except for the two largest biological datasets, 16S.T and 16S.B.ALL, for which we used 346 and 573 bootstrap replicates, respectively.

**Table 1:  Empirical statistics for reference alignments and reference trees for each dataset.** “# Taxa” is the number of taxa. “# Sites” is the number of sites in the reference alignment, which is the curated alignment for the CRW datasets and the true alignment for the Price 78K dataset. “Indels” is the percentage of cells in the reference alignment matrix that consist of indels. “Res” is the resolution of the reference tree, or the percentage of internal edges present in the reference tree out of the total possible number of edges. The p-distance between two sequences is the percentage of sites in which the two sequences have different nucleotides, and is denoted by "p-dist".  “Avg p-dist” is the average p-distance across all pairs of sequences in the reference alignment, and “Max p-dist” is the maximum p-distance across all pairs of sequences in the reference alignment. “Gap Len” is the average length of a gap (contiguous string of indels) in the reference alignment. n=1 for all reported values. 

**Table:** 

	** # Taxa**	** # Cols **	**Res (%) **	** Avg p-dist**	** Max p-dist**	**Indels (%)**	** Avg gap len**
Price 78K	78132	1287	100.0	40.6	64.0	0.6	1.3
16S.B.ALL	27643	6857	17.4	21.0	76.9	80.0	4.9
16S.T	7350	11856	49.9	34.5	90.1	87.4	12.1
16S.3	6323	8716	50.4	31.5	83.3	82.1	9.4
16S.M.aa_ag	1028	4907	42.2	34.2	100.0	82.6	22.0
16S.M	901	4722	46.9	35.9	88.7	78.1	17.2
23S.M	278	10738	61.1	37.7	70.3	83.7	31.9
23S.M.aa_ag	263	10305	60.0	37.7	70.7	83.5	34.2
23S.E.aa_ag	144	8619	64.5	30.3	57.0	61.1	13.5
23S.E	117	9079	65.8	29.6	51.7	59.7	12.6

## 2.3  Methods

### **Alignment methods**

We estimated alignments using SATé, Prank+GT [16], Muscle, Opal, MAFFT, MAFFT-PartTree [30], ClustalW, and ClustalW-Quicktree.  The MAFFT-PartTree and ClustalW-Quicktree, methods are the variants of MAFFT and ClustalW, respectively, designed for use on very large datasets. The commands used for these methods are provided in the Appendix. Due to computational challenges, not all alignment methods could be run on all datasets; details of this are given in Results below.  

    The SATé analysis we performed depended on the dataset size. The default setting for SATé was used on the six smallest datasets. The default version begins by computing four trees, formed by running RAxML to completion on MAFFT, Prank+GT [16], Muscle, and ClustalW alignments. It then takes the tree/alignment pair that had the best ML score as its starting tree, and iterates, alternating between alignment estimation (using a divide-and-conquer technique that constructs alignments on subsets of taxa using MAFFT, and then merges alignments using Muscle or Opal) and tree estimation using RAxML. This iterative stage lasts by default for 24 hours. The running time of SATé thus includes a very expensive initial stage where four different two-phase methods are run, and a potentially expensive second stage if the number of iterations that are run is large.

    We modified this default setting for the four larger datasets, as follows. First, we replaced the expensive initial stage by only computing RAxML on the MAFFT-PartTree alignment. Second, instead of running the next stage for 24 hours, we set the number of iterations in advance. On the 16S.3 dataset we ran SATé for five iterations, and we ran SATé for ten iterations on the 16S.T dataset. We attempted to run SATé on the largest biological dataset (16S.B.ALL, with 27,643 sequences), but it failed to complete its first iteration. We therefore did not attempt to run SATé on the largest dataset (the Price 78K dataset).

**Maximum Likelihood Estimation Methods**

We ran RAxML to compute trees on all but the Price 78K dataset, but tailored the specific RAxML command according to the dataset size; see the Appendix for details. For the Price 78K dataset, we computed the ML tree using FastTree, since our other analyses suggested that the RAxML analysis would require many months to complete.

### 2.4  Measurements

For each alignment produced on each dataset, we used custom code [25] to compute the alignment error using the SP-FN error measure, which is the proportion of the truly homologous pairs of nucleotides (as defined by the reference alignment) that are missing in the estimated alignment [31]. For each tree produced on each alignment, we used custom code [25] to compute the missing branch (or false negative) rate, which is the proportion of the internal branches in the reference tree missing in the estimated tree. We use the missing branch rate instead of the bipartition distance (also known as the Robinson-Foulds (RF) error rate [32]) because the biological reference trees are not completely resolved (high RF rates are always obtained when the reference trees are highly unresolved).

    We also recorded the running time and the memory requirement. For the running time, we report the clock time; this is approximate, since analyses were performed on machines that were not dedicated to these analyses. For the memory requirement, we only report the memory available in the machine on which the method was able to run.

## **3  Results**

### 3.1  Performance on the six smallest datasets

The six smallest datasets, 16S.M.aa_ag, 16S.M, 23S.M, 23S.M.aa_ag, 23S.E.aa_ag, and 23S.E, range in size from 117 taxa to 1028 taxa, and from 4722 sites to 10,738 sites (see Table 1). Thus, none of these is particularly large. On these datasets, all alignment and maximum likelihood analyses succeeded using a dedicated computing core with dedicated access to at least 512 MB and at most 4 GB of main memory (Tables 2 and 3). The Appendix provides further details about the hardware used for these computations. 

    The recorded running times for the alignment methods varied on these datasets (Table 3). The fastest of the alignment methods is the PartTree variant of MAFFT, which completed on these datasets in at most three minutes (most datasets completed in about one minute), and the slowest is SATé.

    Prank+GT tended to be the most computationally intensive of the remaining methods, using several hours on the smallest datasets, on which most of the other alignment methods completed in under an hour. The next most computationally intensive method was Opal. However, all alignment methods (except for Quicktree and PartTree) took several hours on most of these “small" biological datasets. In fact, alignment estimation took more time than maximum likelihood tree estimation on many of these datasets.

    The SP-FN alignment error rates of the different alignment estimators on these smaller biological datasets also varied (Table 2). The alignment estimation methods with the least average SP-FN error were MAFFT and SATé with roughly 23-24% error. The next group, with 27-30% error, was PartTree, Muscle, and Opal. Finally, Prank+GT, ClustalW, and QuickTree had 39-40% SP-FN error. 

     Performance with respect to average missing branch rates showed SATé and MAFFT with the lowest average missing branch rates of 6-8%, followed by PartTree, Prank+GT, Muscle, and ClustalW with average missing branch rates of 13-16%, and then by Opal and Quicktree with 18-19% average missing branch rates. Thus, alignment error, measured using SP-FN, is not particularly predictive of tree error; for example, Opal and Prank+GT change their positions quite dramatically with respect to these criteria.

### 3.2  Performance on the four largest datasets

The four largest datasets consist of three biological datasets ranging in size from 6323 to 27,643 taxa, and having from 6,857 to 11,856 sites; in addition, we have one simulated dataset with 78,132 taxa and 1287 sites. Thus, these four datasets present substantial computational challenges. Table 4 gives the comparison between methods in terms of alignment and tree accuracy, and Table 5 gives the running time of these methods on the large datasets.

    We focus first on the three biological datasets, which range in size from 6323 sequences to 27,643 sequences. Many methods aborted on these datasets: five failures on the 16S.B.ALL dataset (the largest), and three on the 16S.3 dataset. Only two alignment methods completed successfully on the 16S.B.ALL dataset, six on the 16S.T dataset, and four on the 16S.3 dataset. In addition, several methods ran for 35 days on  a machine with 256 GB of main memory without returning an alignment, and are still running ("s.r."): Muscle is still running on all three of the largest biological datasets and Prank+GT is still running on the 16S.T dataset. Thus, these datasets are very difficult for these alignment methods.

    The smallest of these datasets is 16S.3, with 6323 sequences.  Only four methods completed on the 16S.3 dataset, three failures occurred (MAFFT, Prank+GT, and Opal),  and one method (Muscle) is still running. In terms of alignment error, PartTree had less error than CLustalW and Quicktree.  In terms of tree error, SATé had the least error (7%), followed by ML(ClustalW) with 9.29%, and then ML(PartTree)  with 11.83%. ML(Quicktree) had very high error of 31.47%.

    On the next largest CRW dataset, 16S.T (with 7350 sequences), six methods completed,  two methods (Prank+GT and Muscle) are still running, and no failures occurred. In terms of alignment SP-FN error, MAFFT is best, followed by PartTree, then SATé, and then Opal, but all four methods are fairly close in SP-FN error (roughly 31%-39%). ClustalW and Quicktree have much higher SP-FN error rates (56% and 63%).  In terms of tree error, ML(MAFFT) is best (7.29%) followed closely by SATé (7.59%), and then by ML(ClustalW) at 10.21% error. ML(PartTree) and ML(Opal) are next, at 16.73% and 18.62%, respectively. Finally, ML(Quicktree) has the highest error at 34.23%. 

     The largest of these three CRW datasets is the 16S.B.ALL dataset, which contains 27,643 sequences. Only two methods, PartTree and QuickTree, succeeded in producing alignments on the 16S.B.ALL dataset. (Muscle is still running; all other methods have aborted on this dataset.) PartTree's alignment has 41.7% error and QuickTree's alignment has 54.4% error. Maximum likelihood analyses of these two alignments produced trees with high error: 13% for ML(QuickTree) and 32% for ML(PartTree). Running times for Quicktree and PartTree alignment methods were very large–-175 and 262 hours, respectively. The maximum likelihood analyses of these alignments took even longer, 1328 and 1254 hours, respectively. The memory requirements for these methods are unknown, but each failed during an individual run with dedicated access to all 32 GB of main memory on one machine and succeeded with dedicated access to all 256 GB of main memory on another machine.

    A comparison of the alignment methods on the 16S.3 and 16S.T datasets shows that PartTree is extremely fast on these datasets, finishing in 1.3 hours on the 16S.T dataset and in less than an hour on the 16S.3 dataset, and QuickTree is in second place at 41.2 and 20.4 hours, respectively. ClustalW and SATé take much longer: ClustalW uses 506 hours on the 16S.T dataset and 440 hours on the 16S.3 dataset, while SATé takes 1505.8 hours on the 16S.T dataset and 563.2 hours on the 16S.3 dataset. (The difference in running time for SATé on these two datasets is because SATé runs for ten iterations on the 16S.T dataset, but only five iterations on the 16S.3 dataset.) Maximum likelihood analyses of the four alignment methods that completed on these two datasets were also computationally intensive: on the 16S.T dataset, RAxML analyses ranged from 121 to 156 hours (depending on the alignment), while on the 16S.3 dataset, these analyses ranged from 55 to 91 hours. We were able to run SATé successfuly on the 16S.T and 16S.3 datasets using machines with 32 GB main memory available for each run, whereas MAFFT and Opal both were unable to analyze the 16S.T dataset on a machine with 32 GB main memory available for each run and failed on the 16S.3 dataset.

    The Price 78K dataset is the largest of these datasets, and so presents a particularly difficult challenge to the alignment methods. On the other hand, the model condition under which this dataset was generated produced a very low number of indels (0.6% of the true matrix is occupied by gaps, two orders of magnitude smaller than what we see for the biological datasets). Therefore, the Price 78K dataset represents primarily a scalability test – i.e., can the alignment method be run on this dataset?  – rather than a test of accuracy for the resultant alignment. Because of the failures of most alignment methods on the 16S.B.ALL dataset, we only attempted to run the Quicktree and PartTree alignments on the Price 78K dataset. Quicktree failed on this dataset, but PartTree completed. PartTree used 71.5 hours to complete on this dataset (less than it used on the 16S.B.ALL dataset). We used FastTree to compute an ML tree on the PartTree alignment, which produced a tree with 9.14% missing branch rate in 2.9 hours. While this is a fairly high error rate, by comparison, FastTree on the true alignment (which took 3.7 hours to complete) had 8% missing branch rate. Since the true alignment has a relatively low number of sites (1287) for the large number of leaves (78,132), it seems likely that the 8% of the edges missing in the FastTree analysis of the true alignment are weakly supported. Therefore, the FastTree analysis of the PartTree alignment of the Price 78K dataset is actually highly accurate. 

**Table 2:  Comparison of missing branch rates and alignment SP-FN errors on the six smallest datasets.  **Each row gives results for a method, and each column corresponds to a dataset. All analyses succeeded using a dedicated computing core with dedicated access to at least 512 MB and at most 4 GB of main memory. The Appendix provides further details about the hardware used for these computations. Missing branch rates (%) are with respect to the reference tree. Alignment SP-FN errors (%) are with respect to the curated alignment. ML tree estimation was performed using RAxML. 

**Table:** 

	** Missing branch rate (%)**						
Method	16S.M.aa_ag	16S.M	23S.M	23S.M.aa_ag	23S.E.aa_ag	23S.E	Avg error
SATé	5.08	5.70	10.12	10.90	6.59	6.67	7.51
ML(MAFFT)	4.16	5.70	11.90	10.90	7.69	6.67	7.84
ML(MAFFT-PartTree)	10.62	8.79	22.02	20.51	14.29	5.33	13.59
ML(Prank+GT)	7.62	11.40	14.29	19.23	27.47	6.67	14.45
ML(Muscle)	21.48	20.19	14.88	17.31	10.99	6.67	15.25
ML(ClustalW)	12.47	10.93	15.48	16.03	23.08	18.67	16.11
ML(ClustalW-quicktree) ‘	9.47	11.40	20.83	18.59	28.57	24.00	18.81
ML(Opal)	18.94	19.71	22.02	26.92	17.58	8.00	18.86
	** Alignment SP-FN error (%)**						
Dataset	16S.M.aa_ag	16S.M	23S.M	23S.M.aa_ag	23S.E.aa_ag	23S.E	Avg error
SATé	22.72	21.98	29.29	28.42	22.15	21.16	24.3
MAFFT	22.59	21.79	28.61	28.26	19.46	18.48	23.2
MAFFT-PartTree	23.09	27.49	32.11	33.85	20.66	19.65	26.1
Prank+GT	40.73	42.40	44.93	44.06	37.32	35.52	40.8
Muscle	31.14	32.02	34.46	35.59	22.79	21.46	29.6
ClustalW	38.22	42.58	46.25	47.65	29.96	38.54	40.5
ClustalW-quicktree	37.49	40.96	48.38	43.84	26.57	28.07	37.6
Opal	27.71	32.36	32.22	35.01	26.64	21.61	29.3

**Table 3:  Runtimes (in hours) for each method on the smaller datasets.**  Each row lists results for a method, and each column corresponds to a dataset. All analyses succeeded using a dedicated computing core with dedicated access to at least 512 MB and at most 4 GB of main memory. The Appendix provides further details about the hardware used for these computations. ML tree estimation was performed using RAxML. The runtimes given for SATé include the initial phase, which includes the calculation of the four two-phase methods, followed by a 24 hour analysis. We do not divide SATé’s runtime into time used for alignment and tree estimation, but just show the total. 

**Table:** 

Dataset	16S.M.aa_ag		16S.M		23S.M		23S.M.aa_ag		23S.E.aa_ag		23S.E	
	Align	Tree	Align	Tree	Align	Tree	Align	Tree	Align	Tree	Align	Tree
SATé	114.5		106.0		68.9		67.2		61.8		60.0	
ML(MAFFT)	9.2	7.6	5.8	3.6	2.8	2.2	1.7	1.1	0.9	0.4	0.8	0.3
ML(MAFFT-PartTree)	<0.1	9.4	<0.1	6.6	<0.1	1.9	<0.1	1.6	<0.1	0.7	<0.1	0.6
ML(Prank+GT)	15.4	17.1	9.7	12.5	7.6	3.3	5.3	2.6	8.3	0.8	7.4	0.7
ML(Muscle)	9.4	6.1	10.3	7.6	3.1	1.1	2.5	1.6	0.6	0.5	0.4	0.4
ML(ClustalW)	5.6	4.5	6.3	2.5	1.5	0.7	1.3	0.8	2.0	0.4	1.7	0.3
ML(ClustalW-quicktree)	0.9	4.3	0.9	3.6	0.5	1.5	0.4	1.2	0.7	0.7	0.7	0.5
ML(Opal)	3.4	14.3	3.2	8.1	2.8	2.8	2.1	1.9	3.8	0.8	4.1	0.4

**Table 4:  Comparison of missing branch rates and alignment SP-FN errors on the four largest datasets.**  Each row lists results for a method, and each column corresponds to a dataset. Missing branch rates (%) are with respect to the reference tree. Alignment SP-FN errors (%) are with respect to the reference alignment. ML estimation was performed using RAxML on all alignments with the exception of alignments estimated on the Price 78K dataset, for which we used FastTree (we note this by using the prefix “FT:" before the missing branch rate for the Price 78K dataset). “F” indicates that the method aborted on the dataset, and “s.r." indicates that the method was still running on a 256 GB machine, at the time of submission of this manuscript. Entry “n.a.” means "not attempted"; in the case of the results for ML trees produced on estimated alignments, this meant that either we did not attempt to run the alignment, or that the alignment estimation did not complete. Entry “n.c." for alignment SP-FN error means "not computed";  this is given for those datasets that are so large that computing the alignment SP-FN error is infeasible due to computational challenges. The Appendix provides further details about the hardware used for these computations. 

**Table:** 

	**Missing branch rate (%)**			
Method	Price 78K	16S.B.ALL	16S.T	16S.3
SATé	n.a.	F	7.59	7.00
ML(MAFFT)	n.a.	F	7.29	F
ML(MAFFT-PartTree)	FT:9.14	32.19	16.73	11.83
ML(Prank+GT)	n.a.	F	s.r.	F
ML(Muscle)	n.a.	s.r.	s.r.	s.r.
ML(ClustalW)	n.a.	F	10.21	9.29
ML(ClustalW-quicktree)	F	13.07	34.23	31.47
ML(Opal)	n.a.	F	18.62	F
	**Alignment SP-FN error (%)**			
Dataset	Price 78K	16S.B.ALL	16S.T	16S.3
SATé	n.a.	F	36.96	24.93
MAFFT	n.a.	F	30.97	F
MAFFT-PartTree	n.c.	41.73	34.29	22.64
Prank+GT	n.a.	F	s.r.	F
Muscle	n.a.	s.r.	s.r.	s.r.
ClustalW	n.a.	F	56.33	52.04
ClustalW-quicktree	F	54.37	63.03	52.84
Opal	n.a.	F	39.33	F

 **Table 5:**  **Runtimes (in hours) for each method on each of the four largest datasets.**  Each row lists results for a method, and each column corresponds to a dataset. ML tree estimation performed using RAxML for all alignments with the exception of alignments estimated for the Price 78K dataset; we note this by using the prefix “FT:" before the missing branch rate for the Price 78K dataset. “F” indicates that the method aborted on the dataset. “s.r” indicates that the method was still running at the time of submission of this manuscript on a machine with 256 GB main memory. Entry “n.a” indicates a method that we did not attempt to run.  The Appendix provides further details about the hardware used for these computations. 

**Table:** 

Dataset	Price 78K		16S.B.ALL		16S.T		16S.3	
	Align	Tree	Align	Tree	Align	Tree	Align	Tree
SATé	n.a.		F		1505.8	1045.4	563.2	604.7
ML(MAFFT)	n.a.		F		614.7	188.2	F	
ML(MAFFT-PartTree)	71.5	FT:2.9	262.2	1328.1	1.3	126.8	0.7	91.3
ML(Prank+GT)	n.a.		F		s.r.		F	
ML(Muscle)	n.a.		s.r.		s.r.		s.r.	
ML(ClustalW)	n.a.		F		506.0	121.2	440.8	55.8
ML(ClustalW-quicktree)	F		175.6	1254.8	41.2	124.6	20.4	81.5
ML(Opal)	n.a.		F		541.5	509.2	F	

## **4  Discussion**

These analyses show that the choice of alignment method has a very large impact on phylogenetic accuracy. On the datasets with at most (about) 1000 sequences, all the alignment methods can be run, although they differ substantially in terms of alignment and tree accuracy, as well as in terms of computational requirements. More specifically, highly accurate trees and alignments can be computed on the smaller datasets (up to approximately 1000 sequences), provided that the most accurate alignment and tree estimation methods are used (i.e., RAxML on the default MAFFT alignment, or SATé). By contrast, the other alignment methods we tested produced alignments that were much less accurate, so that trees estimated on these alignments also had much higher error rates. Finally, although all methods were successfully able to complete their analyses with at most 4 GB of main memory, many alignment methods took about 10 hours to run on many of these datsets, so that alignment estimation took more time than phylogeny estimation in some cases.

    On the larger datasets, with upwards of 6,000 sequences, the analyses show that most methods are unable to run, even when provided with very large amounts of main memory; instead, only the Quicktree option within ClustalW and the PartTree option with MAFFT were able to complete analyses on all the datasets we studied with 6,000 to 28,000 sequences (although some methods are still running), and PartTree was the only method that succeeded on the largest dataset, with 78,132 sequences. Running times on the largest datasets were substantial, anywhere from several days to several weeks, just to obtain the sequence alignment (i.e., not counting the tree estimation step). Memory requirements were also high, as many methods could not run even when given dedicated access to all 32 GB of main memory on a machine, and had to be run on machines with more memory.

    One of the interesting outcomes of this study is the observation that two methods – PartTree and QuickTree – are both able to analyze many large datasets. Even these methods, however, can be computationally intensive on large datasets. For example, on the 16S.B.ALL dataset, with almost 28,000 sequences, QuickTree used about a week and PartTree used almost 11 days. Unfortunately, neither Quicktree nor PartTree is reliable in terms of alignment accuracy: the ML tree on the QuickTree alignment of the 16S.T dataset had 34% missing branch rate, and the ML tree on the PartTree alignment of the 16S.B.ALL dataset had 32% missing branch rate. Thus, the alignment methods that can be run on large datasets are not reliably accurate, and phylogenies based upon these alignments can have high error.

    Finally, we note that these results show that evaluating alignment accuracy using the SP-FN measure is not necessarily predictive of phylogenetic accuracy. For example, ML trees on Prank+GT alignments have reasonably low missing branch rates although Prank+GT alignments have high SP-FN error, and ML trees on Opal alignments have high missing branch rates, although Opal alignments have low SP-FN error. Hence, evaluations of alignment methods in terms of their consequences for phylogenetic accuracy must be performed with care.

**5  Conclusions**

    This study shows that highly accurate alignments can be estimated when the datasets are small enough (at most a few thousand sequences) and only the best methods are used (e.g., SATé and MAFFT), or when the sequences have evolved under sufficiently low rates of indels. However, under other conditions, such as datasets with tens of thousands of sequences, very few alignment methods can even run at all, even when run on machines with very large memories. Furthermore, the alignment methods that can be run on the very largest datasets (i.e., PartTree and QuickTree) are not methods with high accuracy. While accurate alignments can still be computed using these methods if the datasets have almost no indels, alignments estimated when indels are present will generally be poor. The consequence is that maximum likelihood trees estimated on these alignments will be far from highly accurate. Since maximum likelihood tree estimation has become the dominant method for use in large-scale phylogenetic studies, this study shows that trees estimated on alignments of single genes will be likely to fail to recover many of the well-supported edges of trees estimated on the true alignments.  

    What are the consequences of this study for large-scale phylogenetic estimation? Our study shows clearly that alignments of large datasets that have evolved with many indels will likely have high error rates, and so gene trees estimated from these alignments will also have high error.  At a minimum, this means that the input to species tree estimations (either concatenations of estimated alignments or collections of estimated gene trees) will have high error.  This suggests that  both of the dominant ways of estimating species trees--either  computing trees on concatenated alignments or combining gene tree estimates into a species tree--are likely to have difficulty in producing accurate species tree estimates.  Thus,  the consequence for Tree of Life studies is potentially serious.  

     In summary, the failure of alignment methods to be able to produce highly accurate alignments on large datasets is a limiting factor in phylogenetic estimation, possibly greater than those confronting phylogeny estimation when the true alignment is given. Clearly, new methods need to be developed to enable highly accurate alignment estimation for large datasets, or to construct trees without depending upon multiple sequence alignments of very large datasets.

## **6  Acknowledgments**

 We thank TACC (The Texas Advanced Computing Center) at the University of Texas for assistance in analyzing the 16S.B.ALL dataset.

## **7 Funding information**

  The research was supported by the US National Science Foundation DEB 0733029, and by Microsoft Research through support to TW.

## **8 Competing Interests**

The authors have declared that no competing interests exist.

## **9  Appendix**

### 9.1  Software versions and commands

### Alignment methods

We used ClustalW version 2.0.4, MAFFT version 6.240, Muscle version 3.7, Prank+GT using Prank version 080904, and Opal version 1.0.2, which required Java version 1.6.0_02. 

  In the commands for each method, given below, <input> is a FASTA-formatted input file containing unaligned sequences and <output> is the resulting FASTA-formatted output file.

      • ClustalW: 

      clustalw2 -align -infile=<input>

      -outfile=<output> -output=fasta

      • ClustalW-Quicktree: 

      clustalw2 -align -infile=<input>

      -outfile=<output> -output=fasta

      -quicktree

      • MAFFT L-insi-i: 

      mafft --localpair --maxiterate 1000

      --quiet <input> > <output>

      • MAFFT-PartTree: 

      mafft --parttree --retree 2 

      --partsize 1000 <input> > <output>

      • Muscle: 

      muscle -in <input> -out <output> -quiet

      • Prank+GT: 

      prank -d=<input> -o=<output>

      -t=<RAxML(MAFFT) guide tree> -noxml -notree

      -nopost +F -matinitsize=5 -uselogs

      • Opal: 

      java -jar Opal.1.0.2.jar --in <input>

      --out <output>

### Maximum likelihood estimation methods

For all two-phase methods (i.e., ML trees estimated on alignments other than in SATé), we computed the ML trees using either RAxML version 7.0.4 or FastTree version 2.1.3, but FastTree was used only on the Price 78K dataset. All two-phase ML estimations were run to completion. 

  The commands used are as follows (<input> is a PHYLIP-formatted input alignment file):

      • RAxML default: 

      raxmlHPC -m GTRMIX -w <work dir>

      -n <identifying suffix> -s <input>

      • FastTree: 

      FastTree -nt -gtr -nosupport

      -log <log file> <input alignment> > <output tree>

  The use of RAxML within SATé is also performed as given above, except for the three largest biological datasets. On these datasets (16S.B.ALL, 16S.T, and 16S.3), the SATé search utilized an alpha-version 7.2.4_sse3 of RAxML which makes use of a stopping rule that permits faster runs than the default RAxML version 7.0.4 search.

      • RAxML alpha-version 7.2.4_sse3 fast search, used for SATé: 

      raxmlHPC-SSE3  -F -D -m GTRMIX

      -n <identifying suffix> -s <input>

  Finally, for the purposes of constructing a reference tree on the biological datasets, other than the three largest biological datasets, we used the following command in RAxML version 7.0.4 to estimate an ML tree on the curated alignment and assign support values to the edges of the ML tree using a rapid bootstrapping analysis with 500 bootstrap replicates:

      • RAxML rapid bootstrap: 

      raxmlHPC -f a -m GTRGAMMA -s <input>

      -n <identifying suffix> -x <random number>

      -p <random number> -N <number of replicates>

    To construct a reference tree on the 16S.3 and 16S.T datasets, we first estimated an ML tree on the curated alignment using RAxML version 7.2.6 with the RAxML default command. Then, we performed a rapid bootstrap analysis to assign support values to the edges of the ML tree using RAxML version 7.0.4 with the RAxML rapid bootstrap command. This analysis consisted of 500 rapid bootstrap replicates for the 16S.3 dataset and 346 rapid bootstrap replicates for the 16S.T dataset.

    To construct a reference tree on the 16S.B.ALL dataset, we first estimated an ML tree on the curated alignment using RAxML version 7.0.4 with the RAxML default command. We then assigned support values to the edges of the ML tree using 573 bootstrap replicates from two different bootstrap analyses. 129 out of the 573 bootstrap replicates were obtained using RAxML version 7.0.4 with the RAxML rapid bootstrap command listed above. 444 out of the 573 bootstrap replicates were obtained using RAxML version 7.2.5 to perform a standard bootstrap analysis on TACC's Ranger supercomputer. RAxML was run in parallel on each bootstrap replicate using the four cores of a single compute node on Ranger via the following command:

RAxML analysis of a single standard bootstrap replicate:

 raxmlHPC-PTHREADS-SSE3 -m GTRCAT -s <input> -n <identifying suffix> -b <random number> -p <random number> -N 1 -T 4

  When necessary, we enabled multithreading for the above RAxML runs either by recompiling with PTHREADS [33] and running with an additional flag -T <number of threads>, or by recompiling with MPI for the rapid bootstrapping analysis; this parallelization does not otherwise change the RAxML commands. The RAxML outputs are unaffected by parallelization, and all reported runtimes are for serialized execution.

### SATé estimation

### We ran SATé version 1.1 using the following commands:

• SATé 24-hour search on six smallest datasets:         ./sate_basic.pl -r <name of run>          -w <empty temporary work directory with full path>           -d <input unaligned sequences file with full path>           -l 1 -s -1 -a 5 • SATé search for 10 iterations on four largest datasets:         ./sate_large_64.pl -r <name of run>         -w <empty temporary work directory with full path>         -d <input unaligned sequences file with full path>

### Error calculation software

To compute alignment SP-FN and missing branch error rates, we used custom code, available at the Supplementary Materials online webpage [25].

### 9.2  Computational resources

We ran the two-phase methods and SATé on the six smaller biological datasets using a heterogeneous Condor [10] [34] cluster of machines. Each machine in this cluster has at least 1 core and at most 8 cores on its multi-core processor, which ran at speeds between 1.86 Ghz and 3.16 Ghz. While the machines in this heterogeneous cluster are either 32-bit or 64-bit machines, all machines were capable of running 32-bit executables and we only used 32-bit executables to perform these runs. Each of these runs received its own dedicated core on a machine with dedicated access to at least 512 MB and at most 4 GB of main memory. 

All analyses of the four largest datasets were run using a very high-memory machine with 256 GB main memory and a 16-core 64-bit 2.5 Ghz AMD Opteron CPU, with the following exceptions: each of the successful runs of ML(MAFFT-PartTree), ML(ClustalW-quicktree), and SATé on the 16S.T and 16S.3 datasets and each of the failed runs of ML(MAFFT-PartTree) and ML(ClustalW-quicktree) on the 16S.B.ALL dataset were performed on an individual machine with dedicated access to all 32 GB of main memory and 8-core 64-bit 2.83 Ghz processors. For the runs that failed on the four largest datasets, i.e. SATé and ML(ClustalW) on the 16S.B.ALL dataset and ML(MAFFT), ML(Prank+GT), and ML(Opal) on the 16S.B.ALL and 16S.3 datasets, we performed each run with dedicated access to the entire very high-memory machine and thus each run had exclusive access to all 256 GB of main memory available on that machine. For the runs that succeeded or are still running on the four largest datasets using the very high-memory machine, the minimum amount of memory available to each run was the total amount of main memory available on the machine divided by the number of cores on the machine; however, the memory typically available to each run was much closer to the total amount of main memory available on the machine. 
